# Forest Strata and Abiotic Factors Primarily Regulate Understory Species Richness Rather than Forest Type in a Temperate Forest of South Korea

**DOI:** 10.3390/biology14111565

**Published:** 2025-11-07

**Authors:** Jun-Hyuk Woo, Min-Ki Lee, Jung-Hwa Chun, Chang-Bae Lee

**Affiliations:** 1 Department of Forestry Resources, Kookmin University, 77 Jeongneung Rd., Seongbukgu, Seoul 02707, Republic of Korea; bright27@kookmin.ac.kr (J.-H.W.); where0203@kookmin.ac.kr (M.-K.L.); 2 Forest Carbon Graduate School, Kookmin University, 77 Jeongneung Rd., Seongbukgu, Seoul 02707, Republic of Korea; 3 Forest Ecology Division, National Institute of Forest Science, 57 Hoegi Rd., Dongdaemungu, Seoul 02455, Republic of Korea

**Keywords:** abiotic factors, understory, woody understory plants, herbaceous understory plants, forest strata

## Abstract

**Simple Summary:**

Plants growing beneath the main forest canopy, known as understory vegetation, play a key role in maintaining healthy forests such as habitats for animals, recycle soil nutrients, and enhance the overall diversity of plant life. Despite their importance, most previous studies have mainly focused on how environmental conditions affect these plants. In this study, we examined how abiotic factors (including topography, climate, and soil nutrients) and biotic factors (such as the number and diversity of plants in the overstory and midstory) shape the diversity of understory species across seven forest types. We found that woody understory plants are mainly influenced by the diversity of overstory, whereas herbaceous plants are more strongly affected by soil phosphorus levels. These findings suggest that different processes control woody and herbaceous understory plants. Understanding these mechanisms can help forest managers develop more effective conservation and restoration strategies to maintain plant diversity and ensure the long-term health and stability of forest ecosystems.

**Abstract:**

The understory vegetation forms an important ecosystem by providing habitat, cycling nutrients, and contributing to community diversity. However, previous studies have focused on identifying mechanisms between understory herbaceous diversity and abiotic factors. This study conducted a comprehensive analysis of the effects of abiotic factors (topography, climate, and soil) and biotic factors (species richness and individuals by forest strata), as well as stand age, on understory species richness. Also, we analyzed the effects of seven different forest types in the sampled plots. The most important factors were selected through a multimodel inference test and then applied to piecewise structural equation models on total, woody and herbaceous understory plants. In the total model, elevation-associated temperature had positive effects, respectively. In the woody model, overstory species richness has an indirect positive effect on woody understory plants through the midstory species richness. In the herbaceous model, total phosphorus and elevation-associated temperature had a positive effect on herbaceous understory plants. Therefore, this study indicates that woody species richness controlled by biotic factors and herbaceous species richness controlled by abiotic factors. Our study suggests that woody and herbaceous species richness are regulated by different mechanisms, highlighting the need for distinct management methodologies to enhance plant diversity in forest ecosystems.

## 1. Introduction

Understory vegetation is a major component of the forest ecosystem and is defined as herbaceous and seedlings which are woody plants growing below two meters in the forest [1]. This understory vegetation contributes significantly to the ecosystem functioning through the establishment of microhabitats for organisms including insects and microbes, preventing soil erosion, and regulating nutrient cycling [2]. In particular, the biodiversity of the understory is characterized by complex interactions with understory composition, dominant species, and environmental factors. Therefore, developing models that can predict these interactions is critical to understanding the relationships between forest biodiversity and ecosystem functions [1,2].

In temperate forest ecosystems, on average, herbaceous species account for more than 80% of plant biodiversity, which are responsible for providing stable ecosystem functions and ecosystem services [3,4,5]. Herbaceous leaves, which contain high levels of nutrients, decompose rapidly in the litter layer, thereby contributing to the cycling of nitrogen, phosphorus, and calcium, which are essential elements for trees and plants [3,6]. According to the vernal dam hypothesis, these herbaceous nutrients function by reducing nutrient losses through their capacity to store nutrients during the early spring period [7], and subsequently providing nutrients to trees in the late spring [6]. Furthermore, herbaceous plants have been shown to function as a biological filter for the growth of woody understory plants, thereby influencing competition for resources and habitat [8].

Another component of the understory, the woody understory plant, competes with herbaceous plants for resource availability to enable upward movement [3]. In addition to this extreme competition, seedlings of woody understory plants have poor nutrient availability, making them a vulnerable period in the tree’s life cycle with the highest mortality rate [9]. Therefore, the successful survival of seedlings is of critical importance, as it serves as the foundation for the development and sustainability of the plant community [10].

Two main hypotheses have been developed to explain patterns of species diversity in understory plants: the resource heterogeneity hypothesis and the resource quantity hypothesis. The resource heterogeneity hypothesis suggests that species diversity is regulated by spatial heterogeneity in resource supply and interspecific differences in resource requirements such as light and soil nutrients [11,12,13]. This resource heterogeneity within a given space has been demonstrated to promote niche differentiation, thereby resulting in the settlement of various species even in unfavorable environmental conditions [12,13]. The resource heterogeneity hypothesis predicts species diversity based on the interaction between abiotic factors and resource variability [11,12]. The resource quantity hypothesis is a hypothesis that species diversity is determined by average supply, which is limited by light and nutrients in the understory [14,15].

Previous studies have shown that biotic factors such as the canopy cover of trees and shrubs, as well as forest age, which indicates the stage of forest development, control the species richness of herbaceous plants [16]. In addition, it has been reported that the forest development stage growth determines light availability, microclimate, and soil characteristics, thereby controlling species diversity within forests [17,18,19]. Furthermore, it has been reported that topographical factors, including elevation and slope, exert a significant influence on the quality of water and the characteristics of the soil. These factors, in turn, regulate the species diversity of herbaceous plants [20,21]. The results of these previous studies in forests show that the species richness of understory herbaceous plants is controlled by the complex interaction of biotic and abiotic factors.

Despite numerous previous studies on the species richness of understory plants, these studies have the following clear limitations: (1) Previous studies have focused only on the diversity of herbaceous plants in the understory, and there is a lack of research on the species diversity of woody understory plants [21,22]; (2) Previous studies have focused on the effects of abiotic factors such as light and soil nutrients, and have not explored how biotic factors at different forest levels affect understory species diversity [21,23,24].

**Figure 1:**
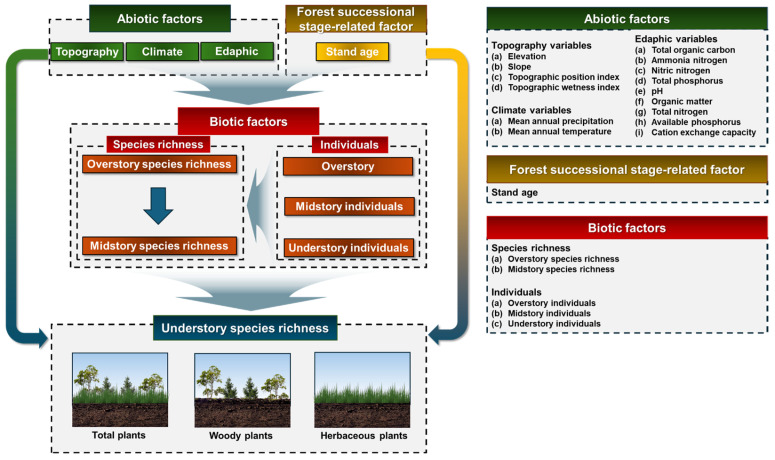
Conceptual model illustrating the hypothesized effects of abiotic, biotic, and forest successional stage-related factors on the species richness of total, woody and herbaceous understory plants in Mt. Gariwang, South Korea.

Based on this conceptual model (Figure 1), we suggest the following research hypotheses: (1) The main factors controlling the mechanism of species richness in herbaceous and woody understory plants are expected to vary; (2) The herbaceous and woody understory plant species will demonstrate different responses depending on the forest types. The objective of this study is to statistically derive factors that significantly influence the abundance of herbaceous and woody understory plants, in order to test this research hypothesis, and to compare the mechanisms that control species abundance patterns between herbaceous and woody understory plants.

## 2. Materials and Methods

### 2.1. Study Area and Data Collection

This study was conducted at Mt. Gariwang, located in Pyeongchang and Jeongseon, Gangwon Province. Mt. Gariwang is classified as a temperate deciduous and mixed coniferous forest biome and belongs to the mountainous ecological region [25]. Additionally, vegetation types vary with elevation, encompassing diverse biogeographic regions. Lowlands feature temperate deciduous and coniferous trees such as *Pinus densiflora* Siebold & Zucc., *Quercus mongolica* Fisch. ex Ledeb., and *Betula costata* Trautv. The subalpine zone (1300 m–1900 m) is distributed *Abies nephrolepis* (Trautv. ex. Maxim.), and *Betula ermanii* Cham. Additionally, rare plants of high conservation value, such as *Thuja koraiensis* Nakai, *Rodgersia podophylla* A. Gray, and *Oplopanax elatus* (Nakai) Nakai, are also distributed in Mt. Gariwang [26]. The mean annual temperature of the plots ranged from 5 °C to 8.8 °C and the mean annual temperature of the plots ranged from 1293 mm to 1758 mm. The elevation distribution of the research area ranged from 621 m to 1470 m.

**Figure 2:**
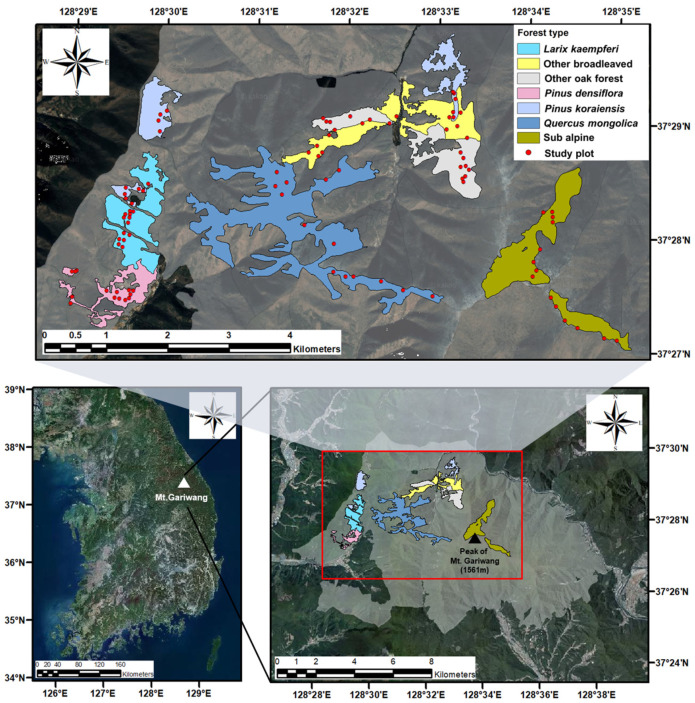
Location and distribution of 98 study plots (400 m^2^) in Mt. Gariwang in South Korea. Red box indicates the main study area within the Mt. Gariwang region.

The study area was divided into seven forest types, considering biogeographical distinctions: three temperate coniferous forest regions, three temperate broadleaf forest regions, and one subalpine region: *Larix kaempferi* (Lamb.) Carrière, *Pinus densiflora* Siebold & Zucc., *Pinus koraiensis* Siebold & Zucc., *Quercus mongolica* Fisch. ex Ledeb., other *Quercus* spp., other deciduous broadleaved, and subalpine coniferous forest. Fourteen square plots (20 × 20 m^2^) were arranged for each forest type, for a total of 98 square plots (Figure 2). Individual of seedlings and herbaceous plants were counted in four subplots (2 × 2 m^2^), which were located at each corner of the square plots (20 × 20 m^2^). For forest stratum classification, woody plants with a diameter at breast height (DBH) of 2 cm or more within the plot were classified by species, and their DBH and height were measured.

### 2.2. Quantification of Abiotic, Biotic, and Forest Development Stage Factors

Climate, topography, and soil factors were used to evaluate the influence of abiotic factors on the species diversity and number of individuals in the understory plants. Climate factors affecting physiological mechanisms such as plant growth and environmental adaptation were based on the mean annual temperature and mean annual precipitation data from digital climate maps produced by the National Forest Science Institute [27]. Topographical factors affecting plant distribution and plant growth were calculated using a digital elevation model to determine elevation, slope, topographical position index, and topographical moisture index [28]. To evaluate the effects of soil chemical properties that influence nutrient utilization and nutrient cycling in plants on the abundance of understory plants, soil samples were collected from four corners and the center of each survey plot, mixed, and analyzed. Soil samples were collected from a depth of 0–30 cm, where roots and available nutrients are concentrated. Total organic carbon (TOC), ammonia nitrogen content, nitric nitrogen, total phosphorus (TP), pH, total nitrogen (TN), available phosphorus, and cation exchange capacity were extracted as soil factors from the analysis results of soil samples [29,30,31].

The stand age, evaluated as a proxy for the forest development stage, was determined by extracting the age class from the forest type maps provided by the Korea Forest Service [32]. Stand age classes are divided into 10-year intervals and measured using core samples from dominant and semi-dominant trees constituting over 50% of the canopy, or through forestry and historical records [33,34].

In order to reduce the degree of collinearity between climatic and topographic factors, principal component analysis (PCA) was performed [16,35,36]. The first axis score of principal components was designated PC_EMM, and their scores were utilized as independent variables, signifying the integrated topographic and climatic factors. The extraction of topographic and climatic factors was conducted using ArcGIS Pro Version 3.2.0.

In order to comprehend the biological relationships that are associated with the composition of the understory species, the forest stratum was divided into overstory, midstory, and understory. Based on DBH, overstory was defined as DBH ≥ 10 cm, midstory as 10 cm > DBH ≥ 2 cm, and understory as DBH < 2 cm. When the DBH is 10 cm or greater, the tree is generally considered to be in a stable state, resistant to external disturbances, and capable of withstanding competition [34,35]. Therefore, woody plants with a DBH of 2 cm or less were considered understory plants, and the stratum was divided into overstory and midstory based on DBH of 10 cm [37]. We divided understory plants into herbaceous plants and woody understory plants to investigate the relationship between species richness and abiotic and biotic factors for each type of understory plant. The classification of herbaceous and woody plants was based on plant growth form (Table S1; Figures S1 and S2). Herbaceous plants included ferns, graminoids (Poaceae, Cyperaceae, Juncaceae), broadleaf plants, and woody understory plants, including trees and shrubs with DBH of 2 cm or less. The identification of all species in each plot and subplot was conducted, and the number of species and the number of individuals (stem density) were calculated for each (Table S2). Plant classification and nomenclature followed the WFO’s plantlist as the standard.

### 2.3. Statistical Analysis

Before conducting statistical analysis, we applied log or square root transformations followed by standardization to all factors to improve normality and linearity (Table S3) [35]. We then applied a generalized least squares (GLS) model to determine whether the variables were affected by spatial autocorrelation [38]. Subsequently, we compared a spatial model, which included the geographic coordinates of each plot as a spatial effect, with a non- spatial model that did not include this spatial effect. The fitness of the spatial and non-spatial models was estimated using the Akaike information criterion (AIC). In this study, the influence of spatial autocorrelation was not confirmed, so geographic coordinates were not utilized in subsequent analyses (Table S4).

In addition, Pearson correlation analyses were conducted on biotic and abiotic factors, and stand age at each stratum to eliminate multicollinearity (Figures S3–S5). Variables with high correlation coefficients (|r| ≥ 0.7) were excluded from the analysis. Furthermore, the variance inflation factor (VIF) was utilized to ascertain the presence of multicollinearity in the multiple regression model [39]. In all models, the VIF values were less than 5, indicating that multicollinearity among the factors did not affect the model results.

Multimodel inference was performed to select significant factors for the species richness of the total, herbaceous, and woody species richness in the understory. The forest types were modelled using a random-effects approach to ascertain the impact of forest types. For each model, the factor with the highest regression coefficient for the dependent variable was selected as the abiotic factor and the biotic factor, respectively, based on the results of the multivariate inference. Furthermore, in order to evaluate the relative importance of each factor, the estimated values of the dependent variable for species richness in the total understory, woody understory plants, and herbaceous plants were calculated as percentages relative to the sum of all variable estimates.

In this study, to overcome the limitations of previous studies, we constructed a conceptual model of the effects of abiotic, biotic and forest development stage factors, and forest cover on understory species richness using structural equation modeling, which is known to be the most suitable analysis for explaining direct and indirect effects between various factors. Three models were constructed by setting the total understory, woody understory plants, and herbaceous plants as dependent variables, respectively, according to plant growth forms. Subsequently, the d-segregation test was employed to identify and remove nonsignificant paths in the hypothesized model. To compare the influence of explanatory variables on the response variable within the structural equation model, standardized path coefficients and the variance of the response variable were calculated. The variance of the response variable was calculated by dividing it into two components: the variance attributable to fixed effects (marginal R^2^_m_) and the variance explained by random effects (forest types; conditional R^2^_c_). The assessment of model fit was conducted by calculating Fisher’s C statistic, *p*-value, and AIC for all models.

Multimodel inference and pSEM analysis were performed using the MuMIn and piecewiseSEM packages in R 4.2.2 [40,41].

## 3. Results

**Figure 3:**
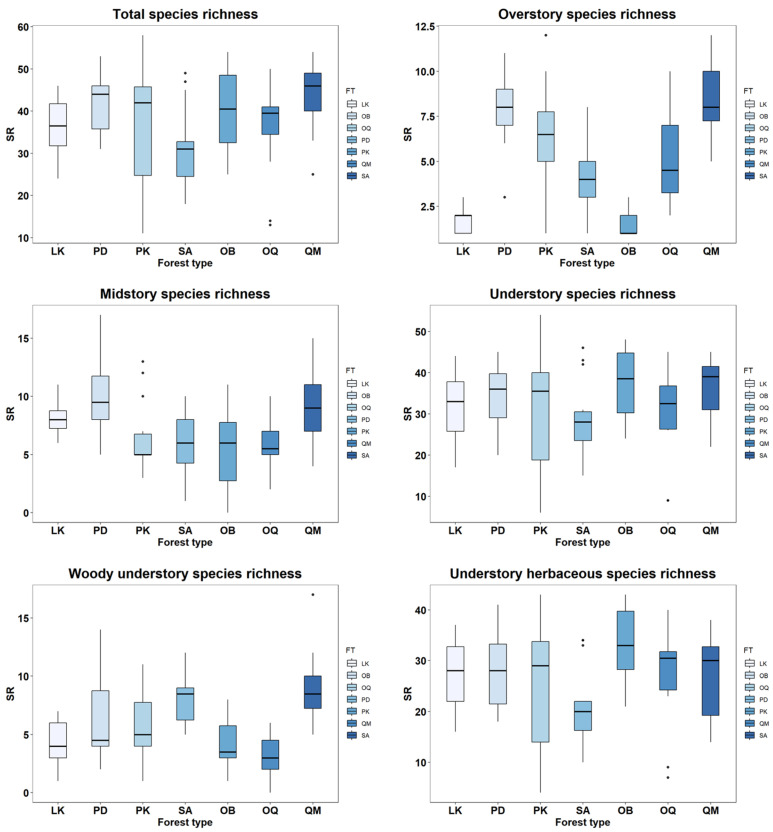
Box plots for the comparison of species richness of total, overstory, midstory, understory (total understory), woody understory, and herbaceous understory plants among forest types. Abbreviation: LK, *Larix kaempferi* forests; PD, *Pinus densiflora* forests; PK, *Pinus koraiensis* forests; SA, subalpine forests, OB; other broadleaved forests; OQ, other *Quercus* forests; QM, *Quercus mongolica* forests.

For Total and Overstory species richness by forest type, it was highest in Korean oak forests and lowest in subalpine forests and other broadleaf forests. Midstory species richness by forest type was highest in pine forests and lowest in Korean pine forests. For understory species richness by forest type and understory herbaceous plant species richness by forest type, it was highest in other broadleaf forests and lowest in subalpine forests. Additionally, understory woody plant species richness by forest type was highest in Korean oak forests and lowest in other oak forests (Table 1 and Figure 3).

**Figure 4:**
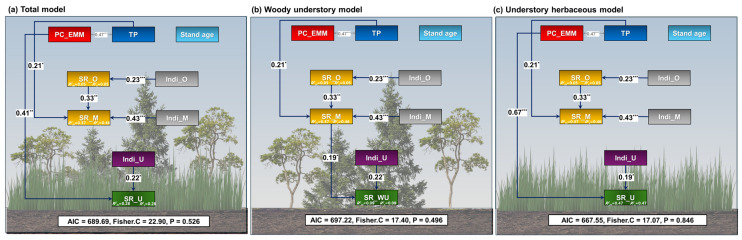
Piecewise structural equation models of total stands accounting for the effects of abiotic and biotic factors and stand age on species richness of (**a**) total, (**b**) woody, and (**c**) herbaceous understory plant in Mt. Gariwang, South Korea. Solid blue and red lines represent positive and negative effects, respectively. The two-way gray arrow indicates the covariance between two variables. Standardized coefficients are shown for each path and covariance. Statistics to evaluate the goodness of fit for models are provided. Abbreviations: AIC, Akaike information criterion; Fisher’s C, Fisher’s chi-square; PC_EMM, the first axis of principal component analysis (PCA) for elevation, mean annual temperature and mean annual precipitation; TP, Total phosphorus; SR_O, Overstory species richness; Indi_O, Overstory individuals; SR_M, Midstory species richness; Indi_M, Midstory individuals, SR_U, Understory species richness; Indi_U, Understory individuals; SR_WU, Woody understory species richness; Individuals_WU, Woody understory individuals; SR_UH, Understory herbaceous species richness; Individuals_UH, Understory herbaceous individuals. Significance levels are indicated as * *p* < 0.05, ** *p* < 0.01 and *** *p* < 0.001.

In the pSEM of the total model (Figure 4a), PC_EMM (β = 0.41, *p* = 0.010) and understory individuals (β = 0.30, *p* = 0.002) were demonstrated to positively influence understory species richness.

The results of the woody understory model (Figure 4b), there was a positive influence of midstory species richness (β = 0.19, *p* = 0.046) and understory individuals (β = 0.23, *p* = 0.017) on understory species richness. Overstory species richness and midstory individuals had a positive effect indirectly through midstory species richness.

**Figure 5:**
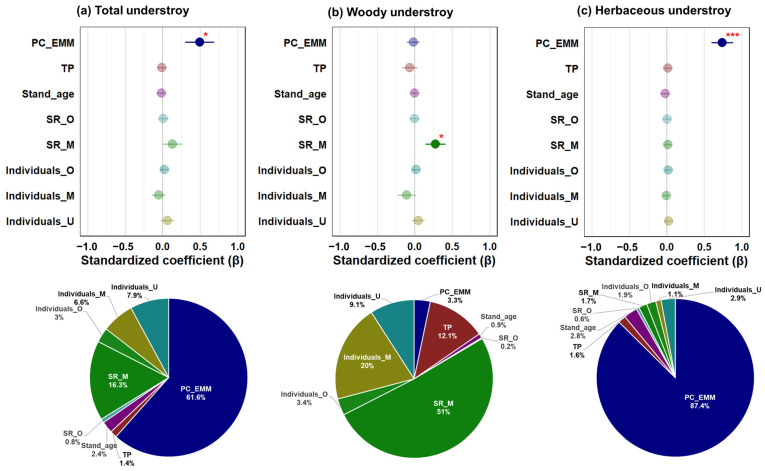
Standardized parameter estimates with error bar of the species richness of (**a**) total, (**b**) woody, (**c**) herbaceous understory plants. The closed and open circles indicate significant and non-significant relationships between species richness and variables, respectively. The relative importance of each factor was calculated as the ratio between the parameter estimate of the sum of all factor estimates and then described as a percentage. All the abbreviations for variables are described in Figure 4. Significance levels are indicated as * *p* < 0.05 and *** *p* < 0.001.

In pSEM of the herbaceous understory model (Figure 4c), herbaceous understory species richness showed a positive relationship with PC_EMM (β = 0.67, *p* < 0.001) and understory individuals (β = 0.19, *p* = 0.019). The results demonstrated that there was no significant difference between R^2^_c_ and R^2^_m_ in all models, thereby confirming that the effect of forest type did not show up in all models. Similar results were shown for bivariate plots and multimodel inference analysis (Figure 5; Figures S6–S8).

## 4. Discussion

Our results suggest that the significant factors and mechanisms controlling species richness of woody understory plants and herbaceous plants are different. The results of this study can be discussed from the following two perspectives: (1) The species richness of woody and herbaceous plants in the understory was not influenced by forest type. (2) The species richness of herbaceous plants in the understory is primarily influenced by abiotic factors. (3) In contrast, the species richness of understory woody plants was more significantly influenced, both directly and indirectly, by biotic factors (species richness in the overstory and midstory, and the individuals of midstory) than by abiotic factors.

In this study, forest type was found to be an ineffective explanatory factor for understory species diversity across all understory plant growth types. This finding suggests that the biodiversity of understory species is primarily determined by abiotic and biotic factors, rather than the forest stand itself. Environmental factors such as soil chemical properties and topographic factors directly regulate understory vegetation growth conditions—such as environmental filtering and soil nutrient utilization—thereby influencing understory species diversity [16,27,42]. Regarding biological factors, high species diversity in the overstory and midstory can induce trait differences between species, thereby increasing understory species diversity. This, in turn, has been shown to increase species diversity in the understory by enhancing the resource utilization efficiency of species, such as their use of light and nutrients [43,44]. These results demonstrate that understory species diversity is more strongly determined by responses to habitat condition regulation by abiotic factors within the forest and biotic factors such as species composition in the overstory and midstory, rather than dependence on specific forest types.

In this study, we found that woody understory plants richness increased with species richness in the midstory. Overstory richness indirectly had a positive effect on woody understory richness through midstory richness. Previous studies have shown that the composition of the overstory and midstory can affect understory species by altering light availability in the understory and increasing soil heterogeneity [45]. So, increasing species richness in the overstory or midstory may suggest that the composition of the overstory changes and that increased heterogeneity increases species richness in the understory by deriving niche partitioning [46]. Moreover, species richness in the overstory and midstory can increase woody understory species richness by providing a variety of seeds.

In this study, total phosphorus had a positive effect on herbaceous species richness. Soil nutrients are a key resource affecting understory richness and diversity [14]. Herbaceous plants were superior in their ability to utilize soil resources compared to woody understory [3,47,48]. For total phosphorus, it is considered an important nutrient in herbs and can reflect the fertility and nutrient status of the soil [42]. In the case of phosphorus, it has a low diffusivity in the soil [23]. This can be explained by the resource quantity hypothesis, whereby limited phosphorus resources with low diffusivity have negatively affected species diversity. Our results are consistent with previous studies, which show that phosphorus has a significant effect on plant species richness in different types of habitats [42].

Herbaceous species richness and PC_EMM showed a positive relationship. Herbaceous species richness increases with increasing elevation and decreasing mean annual temperature. The effect of elevation was consistent with previous studies showing that herbaceous species richness increased with increasing elevation in herbs by a different mechanism than in woody trees [49,50]. This result can be explained by several possibilities. First, many high elevation areas are isolated ‘islands’ of habitat surrounded by inhospitable terrain derived from the presence of abundant snow, ice, rocks and complex aspect. These isolated habitats as refugia for many herbaceous plant species can encourage the evolution (e.g., allopatric speciation) of unique herbaceous species adapted to the specific conditions of the alpine environment [51,52,53]. Second, high elevation areas can contain numerous microclimates within a small geographical area. Variation in topographic characteristics (e.g., slope, aspect) can create microclimates with differing temperatures, moisture levels, and light conditions. These microclimates can provide niches for a wide range of herbaceous plants adapted to specific conditions [53,54]. Third, high elevation area has a short growing season because of the prolonged snow and ice season. Many herbaceous plants fit this condition, as they can produce seeds and complete their life cycles within the brief window of favorable conditions [54]. Moreover, plants in high elevation areas have evolved a range of adaptations to cope with cold temperatures, strong winds, and nutrient-poor soils. These adaptations include dwarf and compact growth forms, deep roots, and specialized mechanisms for cold tolerance, which allow various herbaceous species to thrive in high elevation environments [53,54,55]. However, this phenomenon was caused by the combined effects of the ecological and environmental factors described above, rather than individually.

Available phosphorus also positively impacted native plant cover. Phosphorus is an essential macronutrient, and its soil content is an indicator of soil fertility [22,42,56]. Herbaceous plants, which are generally more efficient at utilizing soil resources than woody plants, can absorb significant amounts of phosphorus [3,48]. This high resource acquisition capacity may enhance herbaceous species richness and distribution in phosphorus-rich soil [42].

Although our study provides significant insights into the relationships between environmental factors and tree diversity in temperate forests, caution is required when generalizing these findings to tropical ecosystems. Differences in climatic regimes, species composition, and disturbance dynamics between temperate and tropical forests may lead to distinct biodiversity–environment relationships [57,58].

## 5. Conclusions

In Mt. Gariwang, the contrasting mechanisms governing species richness patterns between herbaceous and woody understory plants suggest that two plant groups require distinct and separate management strategies. To protect woody understory plants, we should increase species richness in the overstory and midstory, or we should increase the structural diversity of the forest ecosystem through treatments such as thinning and selective cutting to increase resource heterogeneity. In the case of herbaceous plants, there is a way to reduce competition in nutrients by creating an environment for a lot of nutrients or by reducing the population density of herbaceous plants. However, this study did not consider the light factor, which is a key variable addressed in previous studies on understory diversity. Especially, we need to study how light competition affects herbaceous plants growing among large woody plants and perennial trees, such as oak and pine trees. Furthermore, the findings of this study are limited to the local scale of Mt. Gariwang, highlighting the need to account for light-related factors and to investigate the determinants of understory plant diversity across broader spatial extents, such as regional and national scales.

## Figures and Tables

**Table 1:** Species with the highest and second-highest occurrence frequencies at each stratum level across forest types.

Forest Types	Strata	Frequency of Occurrence	
		Most Frequent Species	Second Most Frequent Species
*Larix* *kaempferi* forests	Overstory	*Larix kaempferi* (Lamb.) Carrière	*Fraxinus rhynchophylla* Hance
	Midstory	*Quercus mongolica* Fisch. ex Ledeb.	*Aralia elata* (Miq.) Seem.
	Understory	*Tripterygium wilfordii* Hook.f.	*Rubus pungens* Cambess.
*Pinus* *densiflora* forests	Overstory	*Pinus densiflora* Siebold & Zucc.	*Quercus mongolica* Fisch. ex Ledeb.
	Midstory	*Quercus mongolica* Fisch. ex Ledeb.	*Fraxinus rhynchophylla* Hance
	Understory	*Lindera obtusiloba* Blume	*Parthenocissus tricuspidata* (Siebold & Zucc.) Planch.
*Pinus* *koraiensis* forests	Overstory	*Pinus koraiensis Siebold & Zucc.*	*Fraxinus rhynchophylla* Hance
	Midstory	*Aralia elata* (Miq.) Seem.	*Quercus mongolica* Fisch. ex Ledeb.
	Understory	*Fraxinus rhynchophylla* Hance	*Rubus pungens* Cambess.
*Quercus* *mongolica* forests	Overstory	*Quercus mongolica* Fisch. ex Ledeb.	*Acer pictum* Thunb.
	Midstory	*Acer pseudosieboldianum* (Pax) Kom.	*Symplocos sawafutagi* Nagam.
	Understory	*Pseudostellaria palibiniana* (Takeda) Ohwi	*Ainsliaea acerifolia* Sch.Bip.
Other *Quercus* forests	Overstory	*Quercus mongolica* Fisch. ex Ledeb.	*Acer pictum* Thunb.
	Midstory	*Lindera obtusiloba* Blume	*Acer pseudosieboldianum* (Pax) Kom.
	Understory	*Tripterygium wilfordii* Hook.f.	*Fraxinus rhynchophylla* Hance
Other broadleaved forests	Overstory	*Acer pictum Thunb.*	*Fraxinus rhynchophylla* Hance
	Midstory	*Acer pictum Thunb.*	*Morus indica* L.
	Understory	*Fraxinus rhynchophylla* Hance	*Dryopteris crassirhizoma* Nakai
Subalpine forests	Overstory	*Abies nephrolepis* (Trautv. ex. Maxim.)	*Taxus cuspidata* Siebold & Zucc.
	Midstory	*Acer tschonoskii* Maxim.	*Euonymus macropterus* Rupr.
	Understory	*Carex siderosticta* Hance	*Pseudostellaria palibiniana* (Takeda) Ohwi

## Data Availability

The data that support the findings of this study are available from Chang-Bae Lee, corresponding author, upon reasonable request.

## Supplementary Materials

The following supporting information can be downloaded at: https://www.mdpi.com/article/10.3390/biology14111565/s1, Figure S1: Box plots for the comparison of topography and climate variables among forest types. Abbreviation: LK, *Larix kaempferi* forests; PD, *Pinus densiflora* forests; PK, *Pinus koraiensis* forests; SA, subalpine forests, OB; other broadleaved forests; OQ, other oak forests; QM, *Quercus mongolica* forests; Figure S2: Box plots for the comparison of soil variables among forest types. All the abbreviations for variables are described in Table S3 and Figure S1; Figure S3: Pearson’s correlation coefficient among (**a**) biotic factors and total understory plants, (**b**) topography and climate factors, and total understory plants, and (**c**) soil factors and total understory plants. All the abbreviations for variables are described in Table S3; Figure S4: Pearson’s correlation coefficient among (**a**) biotic factors and woody understory plants, (**b**) topography and climate factors, and woody understory plants, and (**c**) soil factors and woody understory plants. All the abbreviations for variables are described in Table S3; Figure S5: Pearson’s correlation coefficient among (**a**) biotic factors and herbaceous understory plants, (**b**) topography and climate factors and herbaceous understory plants, and (**c**) soil factors and herbaceous understory plants. All the abbreviations for variables are described in Table S3; Figure S6: Bivariate relationships between total understory species richness and all variables. Fitted regressions were significant (*p* < 0.05). All the abbreviations for variables are described in Table S3 and Figure S1; Figure S7: Bivariate relationships between woody understory species richness and all variables. Fitted regressions were significant (*p* < 0.05). All the abbreviations for variables are described in Table S3 and Figure S1; Figure S8: Bivariate relationships between herbaceous understory species richness and all variables. Fitted regressions were significant (*p* < 0.05). All the abbreviations for variables are described in Table S3 and Figure S1. Table S1: List of plant species observed in Mt. Gariwang, South Korea. Abbreviations: LK, Larix kaemferi; OB, Other broadleaf forests; OQ, Other oak forest; PD, *Pinus densiflora*; PK, *Pinus koraiensis*; QM, *Quercus mongolica*; SA, Sub-alpine; Table S2: Summary statistics of all variables across plots (n = 98) in temperate forests of South Korea. Abbreviations: SR, species richness; TPI, topographic position index; TWI, topographic wetness index; MAP, mean annual precipitation; MAT, mean annual temperature; OM, organic matter; TN, total nitrogen; AP, available phosphorus; CEC, cation exchange capacity; TOC, total organic carbon; TP, total phosphorus; AN, ammonium nitrogen; NN, nitrate nitrogen; O, overstory; M, midstory; Min, minimum; Max, maximum; Table S3: The data set used in this study. Abbreviations: SR_O, Overstory species richness; Indi_O, Overstory individuals; SR_M, Midstory species richness; Indi_M, Midstory individuals, SR_U, Understory species richness; Indi_U, Understory individuals; SR_WU, Woody understory species richness; Individuals_WU, Woody understory individuals; SR_UH, Understory herbaceous species richness; Individuals_UH, Understory herbaceous individuals; TPI, Topoghraphic position index; TWI, Topographic wetness index; MAP, Mean annual temperature; MAP, Mean annual precipitation; OM, Organic matter; TN, Total nitrogen; AP, Available phosphorus; CEC, Cation exchange capacity; TOC, Total organic carbon; AN, Ammonium nitrogen; NN, Nitric nitrogen; TP, Total phosphorus.; Table S4: Summary of the generalized least-squares (GLS) models to test spatial autocorrelation. All the abbreviations for variables are described in Table S3.
