# Supplementary material for: Forest Strata and Abiotic Factors Primarily Regulate Understory Species Richness Rather than Forest Type in a Temperate Forest of South Korea
Source: Biology (Basel). 2025 Nov 7;14(11):1565. doi: 10.3390/biology14111565 (PMC12649900; doi:10.3390/biology14111565)
Supplement: Supplementary file 1 [file biology-14-01565-s001.zip › Supplementary material S2 Woo and Lee_11_01_2025.pdf]

Supplementary Material

Forest strata and abiotic factors primarily regulate understory species richness rather than forest type in a temperate forest of South Korea

Jun-Hyuk Woo[1,2], Min-Ki Lee[1,2], Jung-Hwa Chun[3*], and Chang-Bae Lee[1,2*]

[1]Department of Forest Resources, Kookmin University, 77 Jeongneungro, Seongbukgu, Seoul 02707, Republic of Korea

[2]Department of Forestry, Environment, and Systems, Kookmin University, 77 Jeongneungro, Seongbukgu, Seoul 02707, Republic of Korea

[3]Forest Ecology Division, National Institute of Forest Science, Seoul 02455, Republic of Korea

* Correspondence: chunjh69@korea.kr (J.-H.C.); kecolee@kookmin.ac.kr (C.-B.L.)

Table S2. Summary statistics of all variables across plots (*n* = 98) in temperate forests of South Korea. Abbreviations: SR, species richness; TPI, topographic position index; TWI, topographic wetness index; MAP, mean annual precipitation; MAT, mean annual temperature; OM, organic matter; TN, total nitrogen; AP, available phosphorus; CEC, cation exchange capacity; TOC, total organic carbon; TP, total phosphorus; AN, ammonium nitrogen; NN, nitrate nitrogen; O, overstory; M, midstory; Min, minimum; Max, maximum.

Variables	Unit	Total stands			
		Min	Max	Mean	Standard error
Taxonomic diversity					
SR_O	unitless	1	12	5.061	0.319
SR_M	unitless	0	17	7.337	0.323
SR_U	unitless	6	54	32.643	1
Population density					
Indviduals_O	individuals	7	36	18.959	0.595
Indviduals_M	individuals	0	73	26.449	1.433
Indviduals_U	individuals	7	403	124.503	8.812
Forest successional stage					
Stand age	Age class	2	8	5.19	0.162
Topography index					
Elevation	m	621	1470	1021.7	21.352
Slope	degree	3.39	40.32	21.529	0.741
TPI	unitless	-8.75	8	0.494	0.306
TWI	unitless	3.923	11.859	6.148	0.156
Climate index					
MAP	mm	1293	1758	1614.19	11.566
MAT	°C	5	8.8	7.423	0.092
Edaphic index					
pH	unitless	3.4	6.1	5.163	0.041
OM	%	5.88	28.54	10.412	0.283
TN	%	0.282	1.667	0.541	0.019
AP	mg/kg	6.8	175.5	31.564	3.182
CEC	Cmol/kg	17.08	67.21	28.361	0.721
TOC	%	2.75	28.21	6.937	0.301
AN	mg/kg	0	384.6	28.493	4.993
Nitrate nitrogen	mg /kg	0	18.9	2.896	0.414
TP	mg/kg	205.74	1505.33	602.822	25.274

Table S4. Summary of the generalized least-squares (GLS) models to test spatial autocorrelation. All the abbreviations for variables are described in Table S3.

GLS model	Model	Coefficient	t-value	P-value	AIC	R^2_{pseudo}
Total understory						
SR_U ~ PC_EMM	Spatial	0.409	2.623	0.01	257.802	0.066
	Non-spatial	0.402	4.297	< 0.001	271.706	0.161
SR_U ~ TP	Spatial	0.147	1.455	0.149	262.704	0.021
	Non-spatial	0.233	2.352	0.021	283.21	0.055
SR_U ~ Stand age	Spatial	-0.088	-0.894	0.374	264.021	0.008
	Non-spatial	-0.048	-0.473	0.637	288.367	0.002
SR_U ~ SR_O	Spatial	0.317	2.51	0.014	258.674	0.049
	Non-spatial	0.063	0.619	0.537	288.208	0.004
SR_U ~ SR_M	Spatial	0.28	3.258	0.002	255.022	0.096
	Non-spatial	0.22	2.211	0.029	283.828	0.048
SR_U ~ Individuals_O	Spatial	0.104	1.098	0.275	263.748	0.009
	Non-spatial	-0.026	-0.255	0.8	288.526	< 0.001
SR_U ~ Individuals_M	Spatial	0.064	0.675	0.502	264.46	0.004
	Non-spatial	-0.111	-1.091	0.278	267.407	0.012
SR_U ~ Individuals_U	Spatial	0.197	2.275	0.025	260.148	0.049
	Non-spatial	0.283	2.892	0.005	280.574	0.08
Woody understory						
SR_WU ~ PC_EMM	Spatial	-0.23	-1.148	0.254	265.465	0.012

	Non-spatial	-0.203	-2.034	0.045	284.541	0.041
SR_ WU ~ TP	Spatial	0	0.002	0.999	268.069	< 0.001
	Non-spatial	-0.102	-1.009	0.316	287.578	0.01
SR_ WU ~ Stand age	Spatial	0.017	0.174	0.862	268.101	< 0.001
	Non-spatial	0.089	0.874	0.384	287.83	0.008
SR_ WU ~ SR_O	Spatial	0.281	2.166	0.033	263.697	0.032
	Non-spatial	0.04	0.397	0.692	288.433	0.002
SR_ WU ~ SR_M	Spatial	0.297	3.4	0.001	257.54	0.105
	Non-spatial	0.314	3.238	0.002	278.64	0.098
SR_ WU ~ Individuals_O	Spatial	0.098	1.025	0.308	267.173	0.01
	Non-spatial	0.028	0.278	0.782	288.514	< 0.001
SR_ WU ~ Individuals_M	Spatial	-0.002	-0.016	0.987	268.205	< 0.001
	Non-spatial	-0.043	-0.425	0.672	288.41	0.002
SR_ WU ~ Individuals_ U	Spatial	0.302	3.324	0.001	258.014	0.085
	Non-spatial	0.182	1.821	0.071	285.330	0.033
Herbaceous understory						
SR_UH ~ PC_EMM	Spatial	0.675	4.747	< 0.001	227.142	0.157
	Non-spatial	0.618	7.71	< 0.001	242.322	0.382
SR_UH ~ TP	Spatial	0.158	1.776	0.079	240.308	0.033
	Non-spatial	0.349	3.651	< 0.001	276.11	0.122
SR_UH ~ Stand age	Spatial	-0.102	-1.174	0.243	242.062	0.016
	Non-spatial	-0.117	-1.151	0.253	287.275	0.014

SR_UH ~ SR_O	Spatial	0.226	1.968	0.052	239.13	0.036
	Non-spatial	0.049	0.485	0.629	288.356	0.002
SR_UH ~ SR_M	Spatial	0.152	1.953	0.054	239.899	0.037
	Non-spatial	0.059	0.578	0.565	288.257	0.003
SR_UH ~ Individuals_O	Spatial	0.074	0.882	0.38	242.728	0.007
	Non-spatial	-0.05	-0.491	0.625	288.35	0.003
SR_UH ~ Individuals_M	Spatial	0.101	1.227	0.223	242.065	0.014
	Non-spatial	-0.105	-1.035	0.303	287.526	0.011
SR_UH ~ Individuals_U	Spatial	0.252	3.202	0.002	233.896	0.068
	Non-spatial	0.380	4.031	< 0.001	273.574	0.144

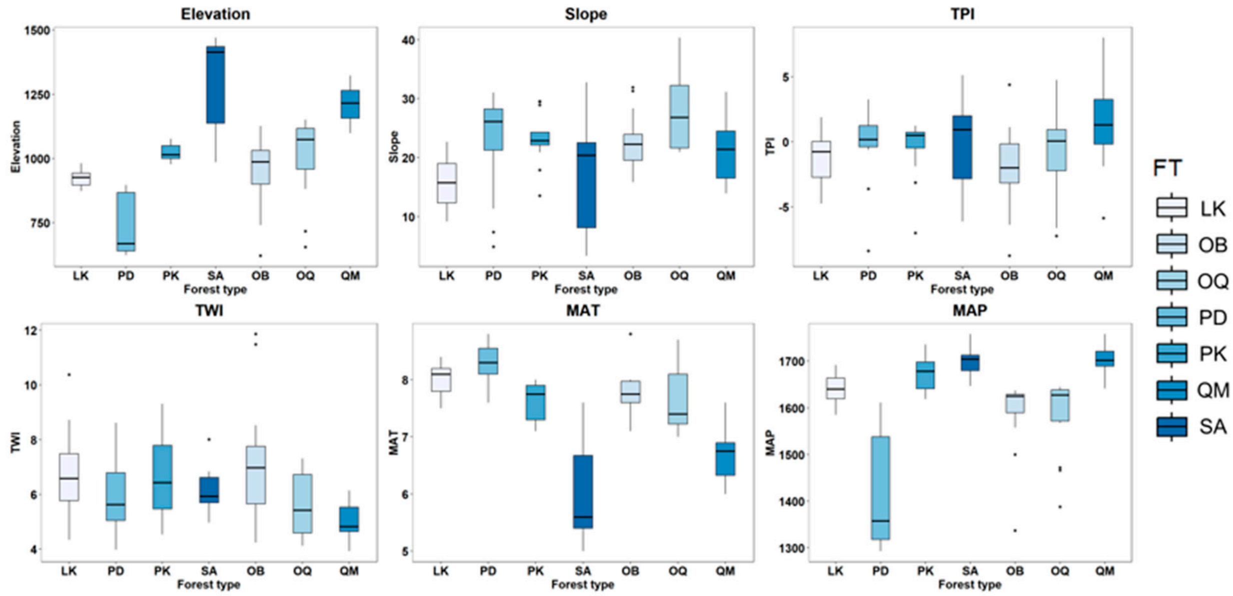

Figure S1. Box plots for the comparison of topography and climate variables among forest types. Abbreviation: LK, *Larix kaempferi* forests; PD, *Pinus densiflora* forests; PK, *Pinus koraiensis* forests; SA, subalpine forests, OB; ordinary broadleaved forests; OQ, ordinary oak forests; QM, *Quercus mongolica* forests.

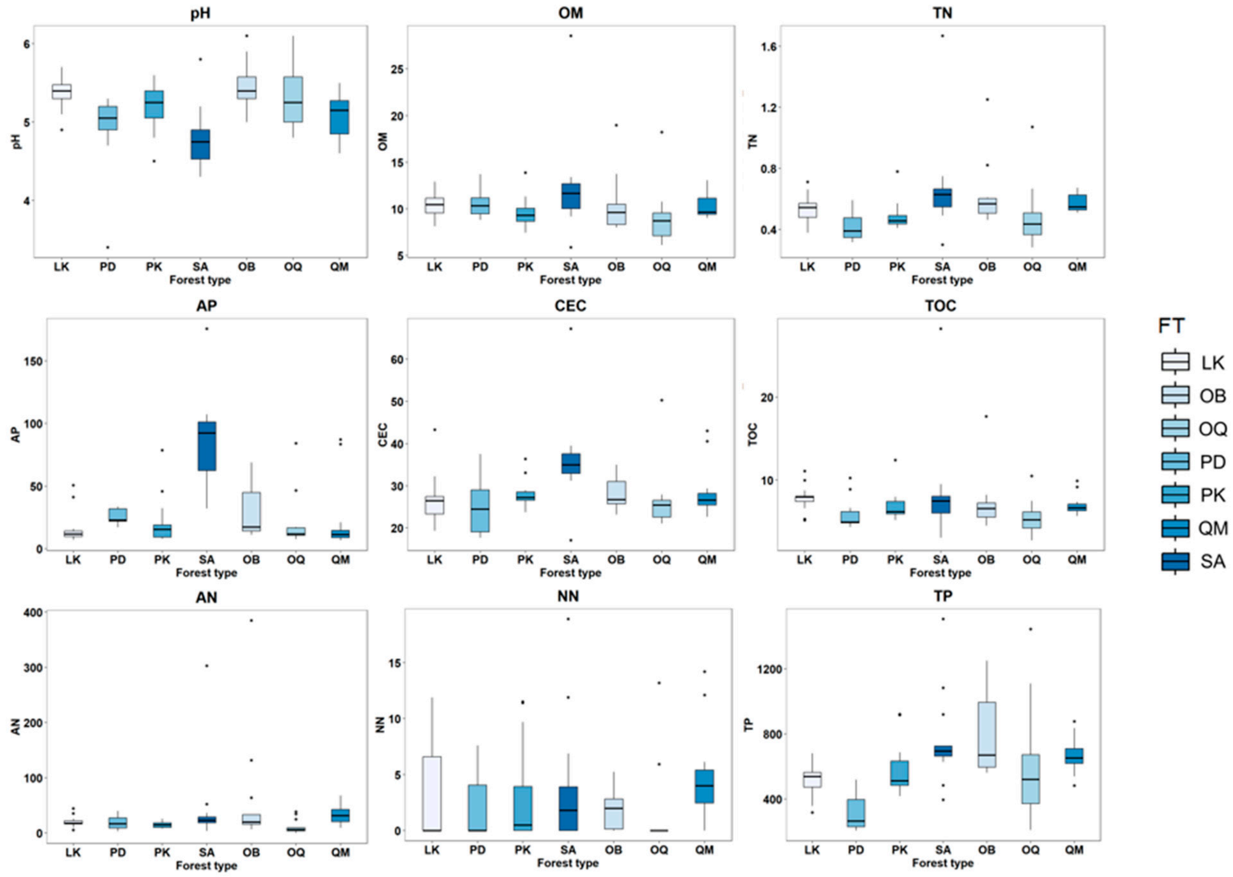

Figure S2. Box plots for the comparison of soil variables among forest types. All the abbreviations for variables are described in Table S3 and Figure S1.

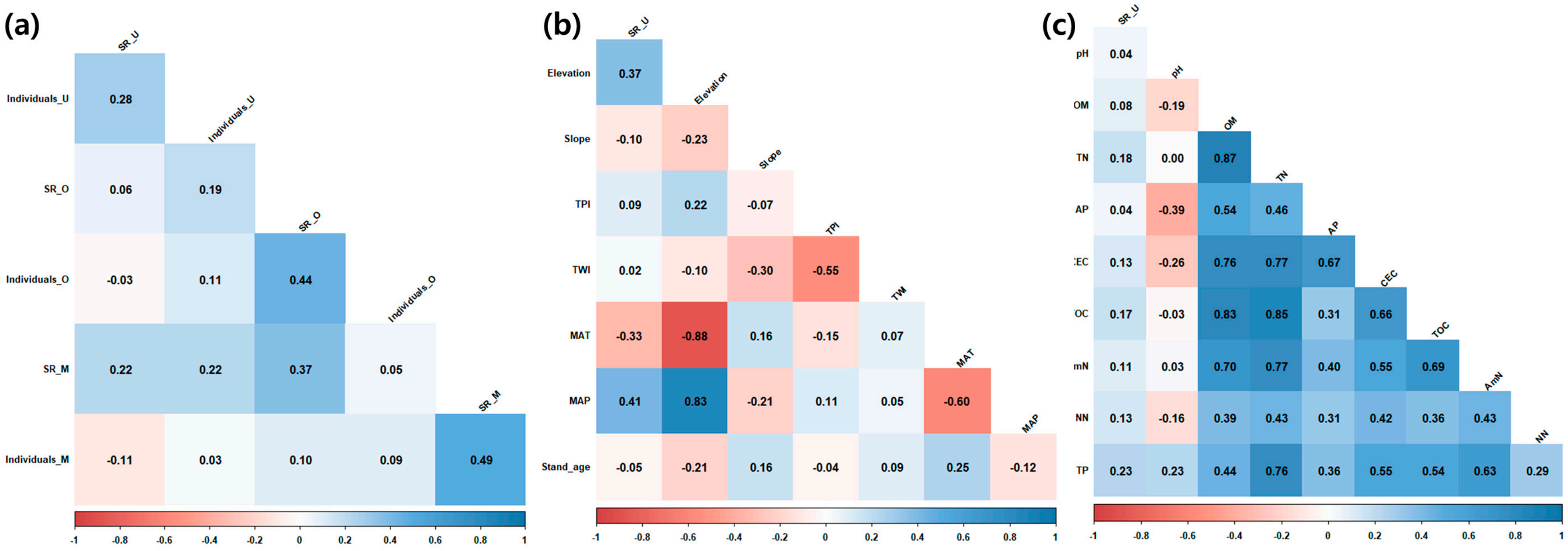

Figure S3. Pearson's correlation coefficient among (a) biotic factors and total understory plants, (b) topography and climate factors, and total understory plants, and (c) soil factors and total understory plants. All the abbreviations for variables are described in Table S3.

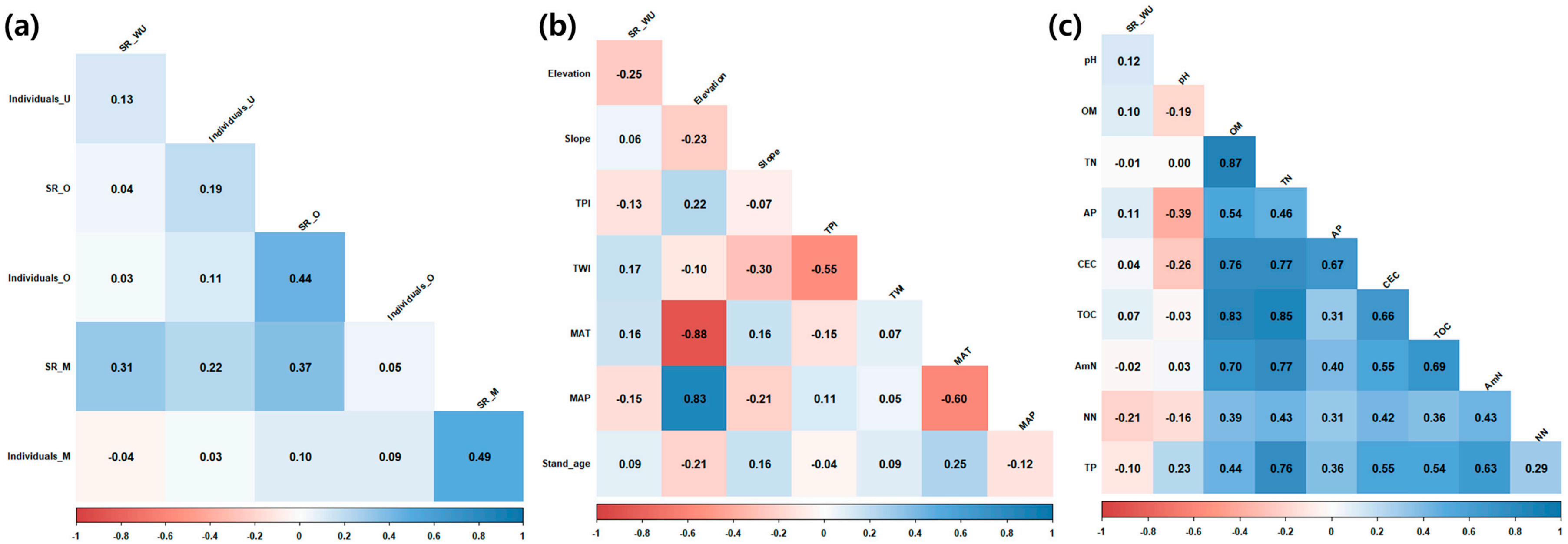

Figure S4. Pearson's correlation coefficient among (a) biotic factors and woody understory plants, (b) topography and climate factors, and woody understory plants, and (c) soil factors and woody understory plants. All the abbreviations for variables are described in Table S3.

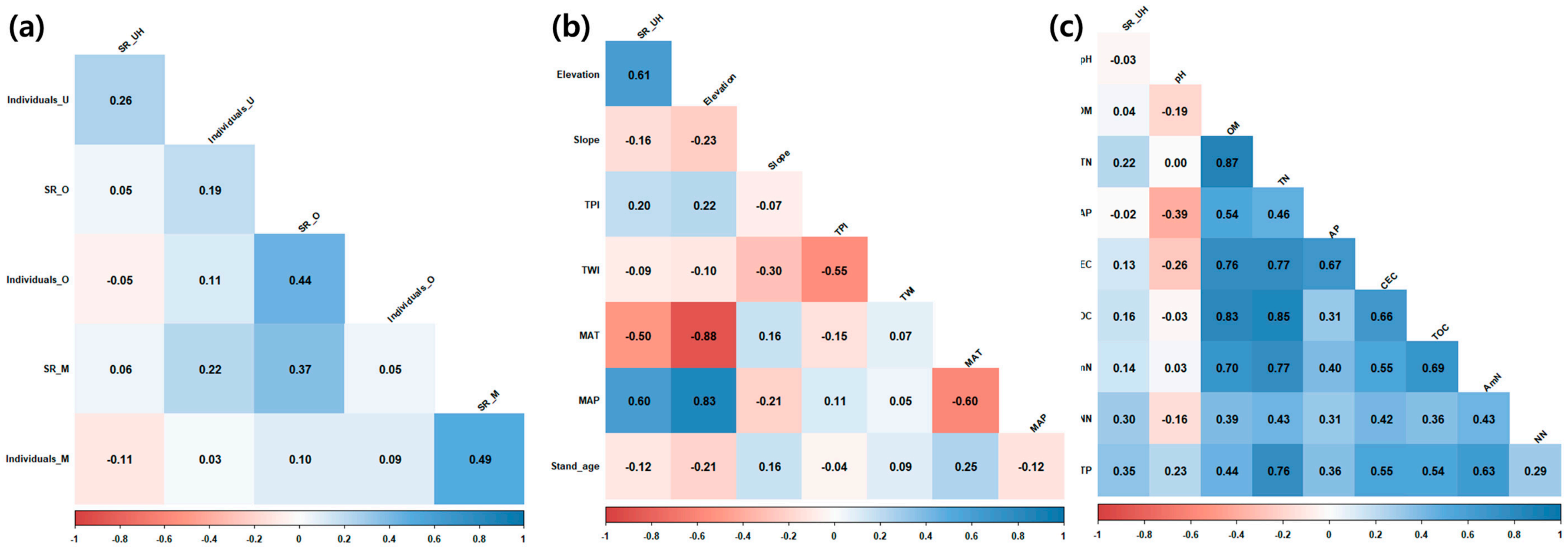

Figure S5. Pearson's correlation coefficient among (a) biotic factors and herbaceous understory plants, (b) topography and climate factors and herbaceous understory plants, and (c) soil factors and herbaceous understory plants. All the abbreviations for variables are described in Table S3.

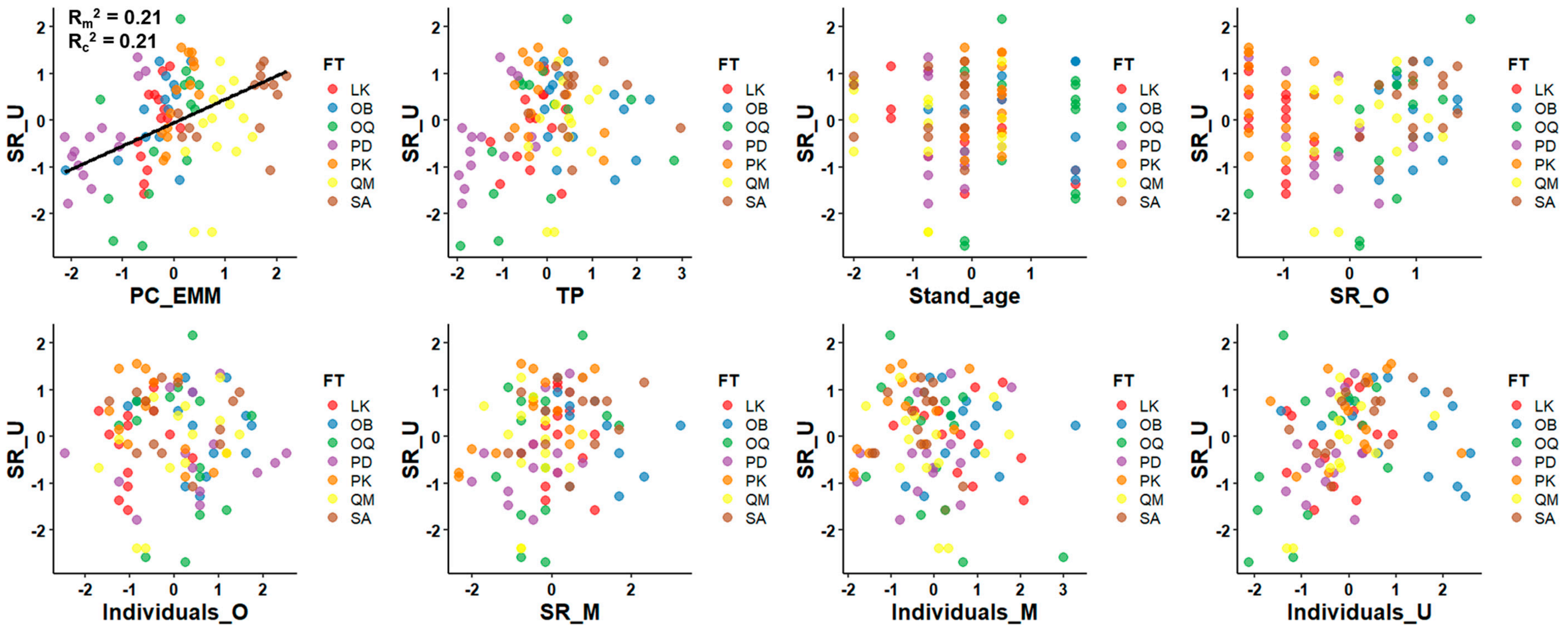

Figure S6. Bivariate relationships between total understory species richness and all variables. Fitted regressions were significant ($P < 0.05$). All the abbreviations for variables are described in Table S3 and Figure S1.

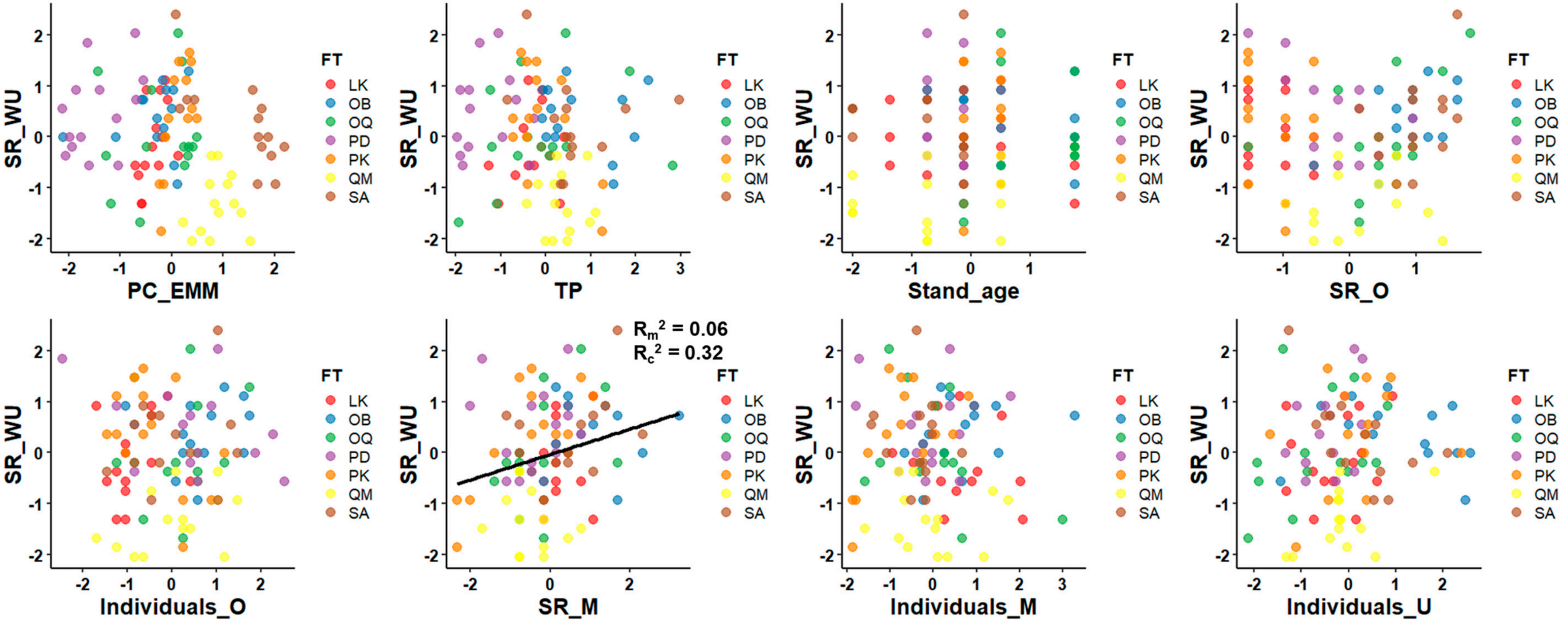

Figure S7. Bivariate relationships between woody understory species richness and all variables. Fitted regressions were significant ($P < 0.05$). All the abbreviations for variables are described in Table S3 and Figure S1.

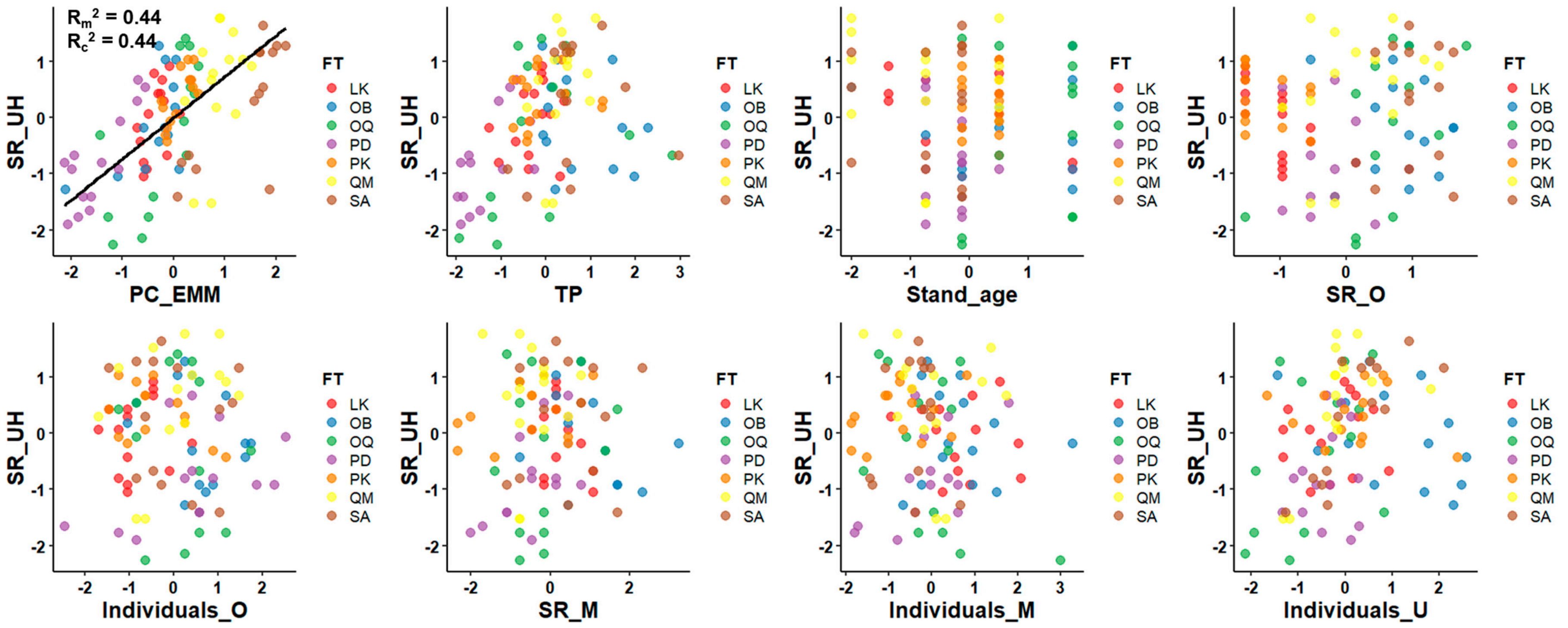

Figure S8. Bivariate relationships between herbaceous understory species richness and all variables. Fitted regressions were significant ($P < 0.05$). All the abbreviations for variables are described in Table S3 and Figure S1.
